# Novel design of (PEG-ylated)PAMAM-based nanoparticles for sustained delivery of BDNF to neurotoxin-injured differentiated neuroblastoma cells

**DOI:** 10.1186/s12951-020-00673-8

**Published:** 2020-08-31

**Authors:** Maria Dąbkowska, Karolina Łuczkowska, Dorota Rogińska, Anna Sobuś, Monika Wasilewska, Zofia Ulańczyk, Bogusław Machaliński

**Affiliations:** 1 Department of Medical Chemistry, Pomeranian Medical University, Rybacka 1, 70-204 Szczecin, Poland; 2 Department of General Pathology, Pomeranian Medical University, Rybacka 1, 70-204 Szczecin, Poland; 3 Jerzy Haber Institute of Catalysis and Surface Chemistry Polish Academy of Sciences, Niezapominajek 8, 30-239 Cracow, Poland

**Keywords:** Brain-derived neurotrophic factor (BDNF), Poly(amidoamine) dendrimers (PAMAM), Neurotoxin-treated neuroblastoma cells, Model of neurodegenerative mechanisms, Nanoparticles encapsulated in polyethylene glycol (PEG), Controlled transport of fragile therapeutic proteins

## Abstract

Brain-derived neurotrophic factor (BDNF) is essential for the development and function of human neurons, therefore it is a promising target for neurodegenerative disorders treatment. Here, we studied BDNF-based electrostatic complex with dendrimer nanoparticles encapsulated in polyethylene glycol (PEG) in neurotoxin-treated, differentiated neuroblastoma SH-SY5Y cells, a model of neurodegenerative mechanisms. PEG layer was adsorbed at dendrimer-protein core nanoparticles to decrease their cellular uptake and to reduce BDNF-other proteins interactions for a prolonged time. Cytotoxicity and confocal microscopy analysis revealed PEG-ylated BDNF-dendrimer nanoparticles can be used for continuous neurotrophic factor delivery to the neurotoxin-treated cells over 24 h without toxic effect. We offer a reliable electrostatic route for efficient encapsulation and controlled transport of fragile therapeutic proteins without any covalent cross-linker; this could be considered as a safe drug delivery system. Understanding the polyvalent BDNF interactions with dendrimer core nanoparticles offers new possibilities for design of well-ordered protein drug delivery systems.

## Introduction

Nanostructures are a promising tool for efficient therapeutics delivery even to the difficult tissues, like brain, in which blood–brain barrier (BBB) remains a fundamental challenge for drug delivery systems. Since brain-derived neurotrophic factor (BDNF) induces neuronal survival and tissue repair, it is a promising therapeutic agent for the treatment of neurodegenerative diseases e.g. Alzheimer’s disease in which cholinergic neurons are depleted [1]; Parkinson’s disease [2] in which dopaminergic neurons of the substantia nigra are lost and amyotrophic lateral sclerosis [3] (ALS) in which cerebral and spinal motor neurons degenerate. However, efficient BDNF delivery to the brain poses a few difficulties.

BDNF is the most abundant member of neurotrophic factors family in the mammalian central nervous system (CNS). BDNF homodimer has about 27 kDa, exerts biological activity in a dimeric state and has a common structural motif consisting of 120 amino acids and forms three disulfide bridges. The isoelectric point of fatty acid free BDNF is pH 10–10.9. The electric charge over BDNF molecules is heterogeneously distributed. As a result, the amino acids structural elements of BDNF molecule like Lysine 96, Arginine 97, Glutamine 84 are presented in the active site, which gives the largest positively charged region over protein molecules. [4, 5] BDNF binds with high affinity to tyrosine kinase B (TrkB) receptor to promote trophic signaling and apoptotic events. [6–8] Indeed, low BDNF levels are observed in brains of patients suffering from multiple pathologies of CNS and changes in BDNF concentration or its distribution have been linked with several neurodegenerative and psychiatric disorders, like depression and schizophrenia. [9, 10].

Neurotrophins are challenging candidates for drug development, because of their low bioavailability for therapeutic targets and insubstantial pharmacokinetic behavior. Synthesis, secreted concentration and half-life of BDNF in human body are limited. [11] BDNF is the diffusible factor secreted by neuronal development system and is not available for all neurons; therefore, its delivery from cells to tissues results in concentration gradients. [12] Accordingly, improved administration of exogenous BDNF and consequent neuroprotection as well as neuroregeneration have been considered potentially novel treatments for neurodegenerative diseases, including Parkinson’s disease. However, carrier-free administration of BDNF is relatively unstable because of rapid degradation in biological medium due to very short in vivo half-life (< 2 min) and low biodistribution causing this side effect [13]. The rational design of BDNF-based nanoparticles requires a good understanding of their interactions for controlled protein release in order to achieve higher delivery efficiency due to increased BDNF presence in the tissue as well as its improved bioavailability.

Nanocarriers such as dendrimer have been extensively studied in various BDNF drug delivery systems [14, 15] to improve their therapeutic efficiency by increasing circulation time and bioavailability at the targeted site (dendrimers possess high biocompatibility and facile functionalization lead to responsiveness to specific stimuli). In our previous studies, on 7th day post application we observed improved administration of neurotrophin-4 using dendrimer nanoparticles in impaired retina tissue. [16] Physicochemical properties of densely branched dendrimer molecules with well-defined spherical geometry, enhance stability and surface functionality of neurotrophin delivery system. The size of dendrimers nanoparticles due to their higher curvature will have a fewer number of nanoparticles ligands that can interact with protein side chains. As the result, PAMAM (poly(amidoamine) dendrimers) surface area accessible to the neurothrophin will be lower, this could potentially result in less protein denaturation. Dendrimers present strong ability to escape from the uptake by non-specific ReticuloEndothelial System and consequently avoid long term toxicities effect [17–20]. In a localized manner, dendrimers, due to the high degree of structural control (monodispersity and tunable chemical structure), can be administrated in vivo as a widely utilized biological functional nanocarriers for drug, [21, 22] biomacromolecule, gene delivery, [23–25] and as imagining agents [26, 27] or diagnostic product [28]. The polyvalent interaction of dendrimers with protein [29, 30] resembles a common type of interaction between biological entities such as receptors and ligands or virus and cell surface, etc. [31–34] Nano-sized carriers systems with dendrimer core are monodisperse therapeutic scaffolds that would possibly allow BDNF delivery to damaged cells, enhancing its local concentration and protein stability against enzymatic degradation. Therefore, we thought to use dendrimers to improve the extracellular retaining of BDNF without the need of covalent chemistry. However, we decided to improve delivery system with PEG [poly(ethylene glycol)], preventing unspecific protein adsorption onto the nanoparticle’s surface [35–37] as well as increasing in vivo blood circulation retention times. Protein resistant PEG layer is independent of the particular choice of PEG molecular weight and thicker polymer brush does not allow proteins to experience electrostatic and van der Waals attractions. PEG brushes are grafted onto dendrimer-protein surface to render them more biocompatible by making nanoparticles less visible to phagocytic cells and improving circulating half-life of nanocarriers. The overall size of negatively charged PAMAM dendrimer 5.5 generation nanocarriers with PEG core could enable efficient diffusion of BDNF across the tissue.

The main goal of our study was to determine efficient encapsulation of BDNF by PAMAM nanoparticles as well as PEG-ylated -PAMAM drug delivery system and assess their usefulness in in vitro system using neurotoxin-treated neuroblastoma model. Therefore, the present study was designed to: (a) elucidate the BDNF desorption from well-characterized PEG-ylated PAMAM dendrimer nanoparticles, (b) investigate diffusion and cytotoxicity of PEG-ylated BDNF-PAMAM dendrimer electrostatic complex in differentiated neuroblastoma SH-SY5Y cells exposed to 6-hydroxydopamine (6-OHDA) in real-time up to 24 h after administration. We used this particular cell line [38–41] and neurotoxin as a model for the degradation of dopaminergic neuron network in the *substantia nigra parts compacta* (SCs) observed in Parkinson’s disease (PD) patients. [42].

## Materials and methods

**Fig. 1:**
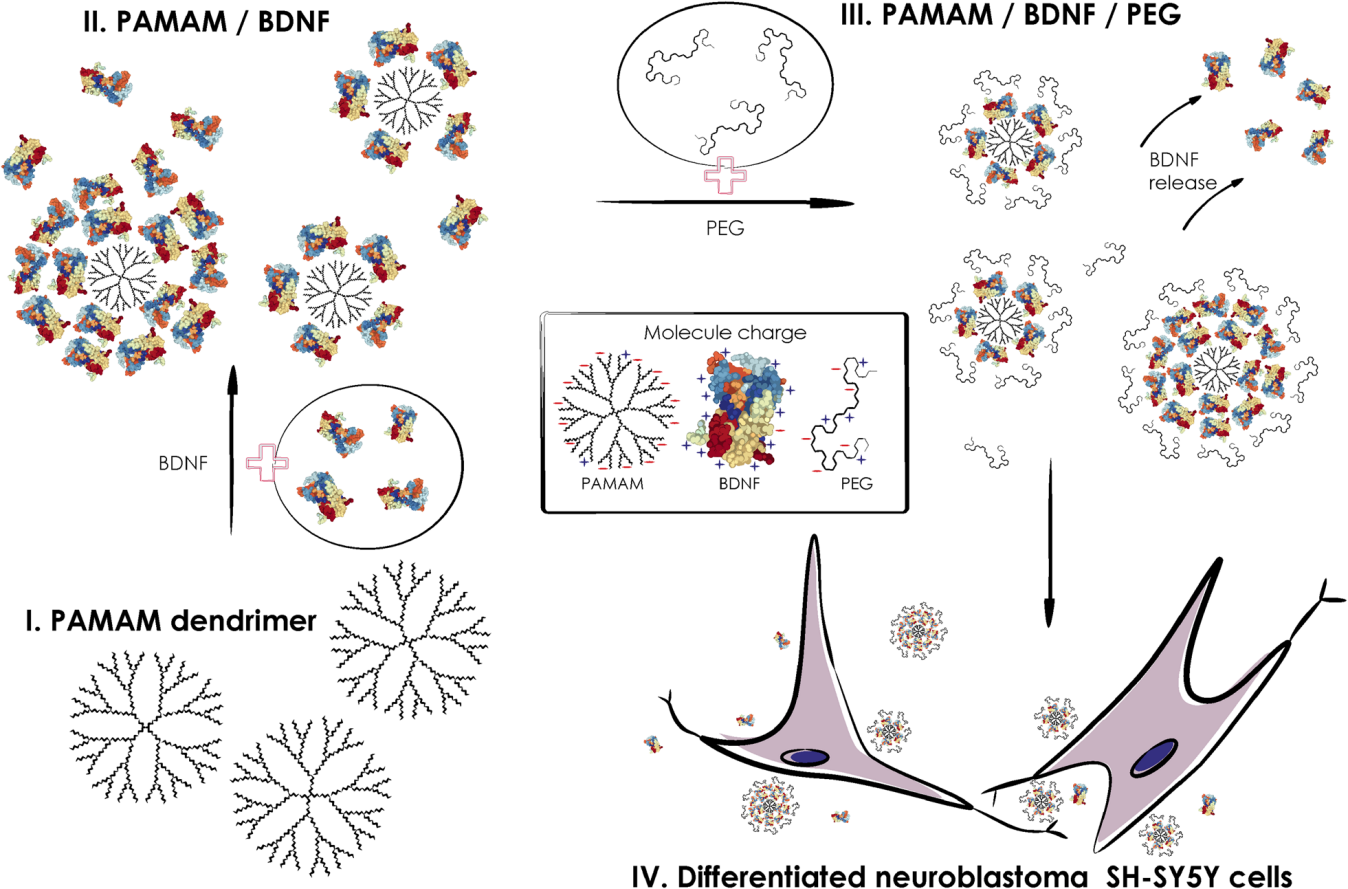
Schematic drawing of PEG-ylated BDNF-PAMAM dendrimer nanoparticles synthesis process as well as BDNF delivery to differentiated 6-OHDA treated neuroblastoma SH-SY5Y cells. Inserted frame comprises molecular structures of BDNF protein, PAMAM and PEG polymer molecules with their overall charge at pH 7.4

Adsorption/desorption transition of BDNF molecules at/from PAMAM dendrimers (Fig. 1) was studied in phosphate buffered saline (PBS) buffer using dynamic light scattering, electrophoresis, solution depletion techniques, enzyme-linked immunosorbent assay and atomic force microscopy. This allowed us to precisely determine maximum loading of BDNF molecule at PAMAM-based nanoparticles under in situ conditions. Afterwards, we compared desorption kinetics of BDNF from PAMAM-based nanoparticles as well as PEG-ylated -PAMAM nanoparticles in PBS buffer and in neuron-like differentiated SH-SY5Y cells treated with 6-OHDA to assess in real time behavior of our nanoparticle in cellular environment (using spectrofluorimetry and confocal microscopy evaluation).

### Nanoparticles synthesis

#### BDNF

Filtered (centrifree ultrafiltration device, Merck Group, Darmstadt, Germany) stock solutions of carrier free recombinant human BDNF (248-N4-250/CF, R&D Systems, Canada) of known concentrations (typically 250 mgL^−1^) in the phosphate buffered saline (PBS) pH 7.4 ± 0.2, 0.15 M (Biomed, Lublin, Poland) were prepared to remove aggregates and provide constant, free form protein molecules concentration in the solvent. To minimize errors in concentration measurements, two complementary spectrophotometric techniques were used: the BCA (protein quantification bicinchoninic acid assay, kit for low concentration, Abcam, Cambridge, UK) and UV absorbance at 280 nm measured with microplate spectrophotometer (BioTek Epoch, United States). Prior to each measurement, the stock solution was diluted to a desired bulk concentration, typically 0.01–2 mgL^−1^. The exact concentration of these solutions after membrane filtration was determined by commercially available Enzyme-Linked Immunosorbent Assay (ELISA) (DY992, DY990, DY994, DY999, DY995, WA126, DY006, DY268, R&D Systems). The temperature of experiments was kept at a constant value equal to 298 ± 0.1 K.

#### PAMAM

The suspension of PAMAM G5.5 ethylenediamine core five and a half generation dendrimers with sodium carboxylate surface groups, (536784, Sigma Aldrich, St. Louis, MO, USA) was used as a colloid carrier for BDNF. The stock suspension was diluted prior to each adsorption experiment to a desired mass concentration, equal to 10 mgL^−1^.

#### PAMAM-AF488 conjugates

For the detection of protein–PAMAM nanoparticles in differentiated neuroblastoma SH-SY5Y cells by confocal microscopy, a detectable fluorescent molecule was necessary. We used Alexa Fluor 488® (AF 488)-NHS esters (Invitrogen™, Thermo Fisher, Waltham, MA, USA) ester as fluorescent label. PAMAM at 5 mg mL^−1^in pH 8.0 sodium bicarbonate buffer reacted with 1 mg mL^−1^ amine reactive dye dissolved in dimethylformamide as described in the manufacturer’s protocol, to give fluorescently labeled PAMAM-AF488 conjugates. These conjugates were purified by extensive dialysis against pH 7.4 to remove unreacted label. After dialysis, we further purified conjugates through centrifugal filtration until filtrate absorbance at 490 nm reached background levels.

#### BDNF-PAMAM dendrimer nanoparticles

BDNF adsorption at PAMAM dendrimers was performed employing electrostatic interactions according to the following procedure: (1) the reference electrophoretic mobility of bare PAMAM nanoparticles was measured, (2) BDNF layers were formed by mixing equal volumes of its solutions of the bulk concentration (varied between 0.002 – 0.4 mgL^−1^), with nanoparticle suspension of the bulk concentration 20 mgL^−1^, (3) the electrophoretic mobility of BDNF-PAMAM nanoparticles was measured and the corresponding zeta potential was calculated. Experiments were conducted at 7.4 pH, ionic strength 0.15 M, 20 °C temperature. The whole experimental procedure was performed with 0.15 M ionic strength, as the adsorbed amount of protein increases proportionately with increasing ionic strength due to the reduction of the repulsive interactions between the protein and dendrimers [16, 43]. In all cases, for adsorption time of 2000s BDNF should be irreversibly adsorbed onto the surface creating monolayer as a result of short-range electrostatic interaction between BDNF and PAMAM. The time scale for formation of additional layers was much longer than that of a monolayer formation. The adsorption time of BDNF at PAMAM dendrimers was prolonged up to 6 h in the primary adsorption experiments. To determine unbounded protein concentration after adsorption, we performed ultrafiltration with a 50 kDa cutoff membrane (Millipore, Billerica, MA, USA), allowing the separation of BDNF-PAMAM (more than 80 kDa) complex and BDNF molecule (28 kDa). Protein concentrations were determined using ELISA protein assay.

#### PEG-ylated BDNF-PAMAM dendrimer nanoparticles

PEG (poly(ethyleneglycol)) with molecular weight of 4 kDa, (1546569, Sigma Aldrich) was used for encapsulation of BDNF-PAMAM dendrimer nanoparticles. PEG chains were conjugated to the nanoparticle surfaces via amide bonds formation between PEG amino groups and PAMAM carboxyl groups. An equal volume of beforehand prepared BDNF-PAMAM and PEG (50 mgL^−1^, pH 7.4, PBS) solutions was stirred at room temperature for 1 h. Further, PEG-ylated BDNF-PAMAM solution was ultrafiltered with a membrane of 10 kDa cutoff (Millipore, Amicon) to remove unconjugated PEG chains.

### Determination of nanoparticle size distribution

#### Bulk

For size determination of BDNF, PAMAM 5.5, BDNF-PAMAM and PEG-ylated BDNF-PAMAM, a dynamic light scattering (DLS) was used.

Suspensions of nanoparticles as well as protein diluted with PBS buffer to a suitable concentration were measured in Zetasizer Nano ZS apparatus (Malvern Instruments, Malvern, UK) equipped with a laser of 633 nm wavelengths. Data analysis was performed in automatic mode at 25 °C. Measured size was presented as the average value of 20 runs, with triplicate measurements within each run. Particle size distributions were obtained from measured diffusion coefficients.

#### Surface

Ruby muscovite mica (Continental Trade, Poland) was used as a substrate for BDNF and PAMAM-BDNF based nanoparticles adsorption measurements. Fresh, solid pieces of mica were cleaved into thin sheets prior to each experiment.

The AFM (atomic force microscopy) technique was used to obtain information about the size distribution of BDNF, PAMAM dendrimers and nanoparticles. The nanoparticle as well as BDNF protein were left to deposit on mica sheets placed in the diffusion cell over a controlled time, and then substrate was removed and rinsed for half an hour in ultrapure water. The samples were left for air-drying until the next day. Next, the dry sample was placed under 7–10 nm AFM tip. The AFM measurements were carried out under ambient air conditions using the NanoWizard AFM (JPK Instruments AG, Berlin, Germany). The intermittent contact mode images were obtained in the air, using ultrasharp silicon cantilevers (NSC35/AlBS, MicroMash, Spain) and the cone angle of the tip was less than 20°. The images were recorded at the scan rate of 1 Hz for the six randomly chosen places. The images were flattened using an algorithm provided with the instrument. We captured all images in random areas within the scan size of 0.5 × 0.5 µm or 1 × 1 µm. BDNF, PAMAM 5.5, BDNF-PAMAM and PEG-ylated BDNF-PAMAM surface dimensions were determined using ImageJ software by gathering the number and coordinates of single protein/nanoparticles molecules. Manual counting of protein/nanoparticles molecules was based on comparing the original image and the same picture altered by digital image filters by cutting off the picture background.

### Nanoparticle zeta (ζ) potential determination

The electrophoretic mobility of BDNF molecules, PAMAM, BDNF-PAMAM as well as PEG-ylated BDNF-PAMAM nanoparticles was measured at pH 7.4 and 0.15 M ionic strength with Laser Doppler Velocimetry (LDV) technique with the aid of the abovementioned Malvern device. LDV method has been introduced by Adamczyk et al. and is based on the measurement of ζ-potential/microelectrophoretic mobility changes during adsorption of tested protein on a model colloid particle. Electrophoretic mobility was recalculated to ζ-potential using Henry equation valid for higher ionic strength where the polarization of the electric double layer is relevant (the double-layer thickness becomes smaller than the protein dimension).

### Cell culture and differentiation

SH-SY5Y neuroblastoma cells (human, ECACC; Sigma Aldrich, St. Louis, MO, USA) were used in this study. SH-SY5Y cells were incubated in culture plates in proliferation medium containing Ham’s F-12 Nutrient Mixture (Thermo Fisher, Waltham, MA, USA) and minimum essential medium (MEM) (Sigma Aldrich, St.Louis, MO, USA) mixed in ratio 1:1 and supplemented with streptomycin (100 µg/mL), penicillin (100 U/mL), L-glutamine (2 mM) and 15% heat-inactivated fetal bovine serum (FBS) at 37 ºC in saturated humidity atmosphere containing 5% CO_2_. The proliferation medium was changed every 2–3 days, and the cells were passaged when they reached 80% confluence. After the proliferation step, the cells were transferred into new culture plates and incubated for 24 h with MEM supplemented with penicillin (100 U/mL), streptomycin (100 µg/mL), L-glutamine (2 mM) and 1% FBS. On the next day, the medium was changed to a differentiation medium consisting of MEM supplemented with penicillin (100 U/mL), streptomycin (100 µg/mL), L-glutamine (2 mM), 1% FBS and Retinoic Acid (0.01 µmol/mL) (RA, Sigma Aldrich, St.Louis, MO, USA). The differentiation was carried out for 5 days and the medium was changed every 2 days.

### Nanoparticles cytotoxicity

Differentiated SH-SY5Y cells were incubated at a density of 3 × 10^4^ cells/well in 96-well plates for 24 h with MEM (without FBS) containing 6-hydroxydopamine (100 µmol/L) (6-OHDA, Sigma Aldrich, St.Louis, MO, USA). 6-OHDA remains the most widely used neurotoxin in Parkinson’s disease (PD) in vitro models [44], due to its structural similarity to dopamine (DA) and high affinity for the DA transporter, which enable it to selectively destroy dopaminergic neurons [45]. Therefore, for our study we chose to treat human neuroblastoma cell line SH-SY5Y with 6-OHDA, as it has been extensively described in the literature as a proper in vitro model for PD [46, 47]. Then, the cells were incubated for 24 h with different concentration of BDNF, BDNF-PAMAM dendrimer nanoparticles or PEG-ylated BDNF-PAMAM dendrimer nanoparticles, adding 20 µl to each well of selected solution prepared in PBS (described in 2.1. Nanoparticles Synthesis) and 80 µl MEM without FBS. The last step was to measure toxicity using the MTT assay (Abcam, Cambridge, UK), which is based on the conversion of water soluble 3-(4,5-dimethylthiazol-2-yl)-2,5-diphenyltetrazolium bromide to an insoluble formazan product, which has a purple color. Cells were incubated with 50 μL of MTT reagent mixed with 50 µl MEM for 3 h, then 150 µl of detergent solution was added to solubilize the colored crystals. Finally, absorbance was measured at OD590nm using Varioskan LUX Multimode Microplate Reader (Thermo Fisher, Waltham, MA, USA). Toxicity was calculated from the equation provided in the manufacturer's protocol.

### BDNF quantification

#### In PBS

The protein release kinetics from PAMAM as well as PEG-ylated PAMAM nanoparticles was assessed by using ultrafiltration method with a 30 kDa cutoff membrane (Millipore, Billerica, MA, USA) in PBS at pH 7.4 and 0.15 M ionic strength. It was done in two-stage procedure, where first BDNF adsorption process was carried out for 1 h. The BDNF molecules released from nanoparticles were quantified with ELISA immunoassay method according to the manufacturer’s protocol. Initially, the residual (unbound) BDNF concentration in the filtrate was determined immediately after adsorption at PAMAM nanoparticles by applying sandwich ELISA technique to monitor simultaneously the maximum concentration of unbound BDNF in the supernatant suspensions. Thus, it was possible to precisely determine concentration of non-adsorbed BDNF molecules at PAMAM as well as PEG-ylated PAMAM nanoparticle surface. These measurements were utilized for determining the maximum coverage of neurotrophin under various protein bulk condition (0.002–1 mgL^−1^). Afterward, the concentration of desorbed BDNF for a different time increment (20 min, 2, 3, 5, 8, 10, 24 h) was quantified with UV–VIS spectroscopy and calculated according to ELISA standard curve.

#### In neuroblastoma cell culture

Concentration of released BDNF molecules was determined by exposing differentiated human neuroblastoma cells SH-SY5Y to 6-hydroxydopamine (6-OHDA) as well as nanoparticles with different BDNF concentrations for 24 h at 37 °C. At the end of the treatments /incubation, the medium was discarded and collected to quantify BDNF concentration using UV–VIS spectroscopy calculated according to ELISA standard curve.

### PAMAM-based nanoparticles behavior in cell culture

In addition, we further investigated the behavior of our nanoparticles loaded with BDNF in SH-5YSY cell culture by determination of green fluorescence of PAMAM-AF488 conjugates. Differentiated SH-SY5Y cells and previously treated with 6-OHDA (protocol described in 2.5. Nanoparticles Cytotoxicity) were incubated at a density of 3 × 10^4^ cells/well in 96-well plates with BDNF-PAMAM-AF488 and BDNF-PAMAM-AF488-PEG nanoparticles (0.1 μg/mL protein loading) for different time lengths (2, 5, 10, 30 min, 1, 4 and 24 h) and were subjected to examinations by spectrofluorimetry evaluation using Varioskan LUX Multimode Microplate Reader.

### Immunofluorescent labeling- nanoparticles imaging in vitro

For immunofluorescence analysis SH-SY5Y cells were plated in 4-well chamber slides at a density of 5 × 10^4^ cells/well. After the differentiation and 6-OHDA treatment (protocols described in Sects. 2.4 and 2.5, respectively) cells were incubated with BDNF-PAMAM-AF488 and BDNF-PAMAM-AF488-PEG nanoparticles (0.1 μg/mL protein loading) for different time lengths (5, 10, 30 min, 1 and 24 h). At designated time points, cells were washed with PBS and fixed with 70% ethanol for 15 min. To visualize surface glycoproteins, cells were stained with wheat germ agglutinin conjugated to Texas Red-X (WGA-Texas Red-X, Thermo Fisher, Waltham, MA, USA) in HEPES buffer for 30 min. After DAPI counterstain, the slides were mounted and subjected to z-stack analysis using a LSM700 confocal system (Carl Zeiss, Jena, Germany).

### Statistical analysis

All presented data are expressed as means ± standard deviation (SD) from at least three independent experiments. Statistical analysis among each study group was performed using Kruskal–Wallis test. Two-Way ANOVA was used for analysis between experimental groups. *p* < 0.05 was considered statistically significant.

## Results and discussion

### Characterization of PAMAM-based nanoparticles

PAMAM-based nanoparticles were prepared based on a modified method previously described in the literature. The prepared BDNF-PAMAM as well as PEG-ylated BDNF-PAMAM nanoparticles were physicochemically characterized in terms of size, polydispersity index and electrophoretic mobility/zeta potential in PBS buffer without calcium and magnesium ions, pH 7.4.

**Fig. 2:**
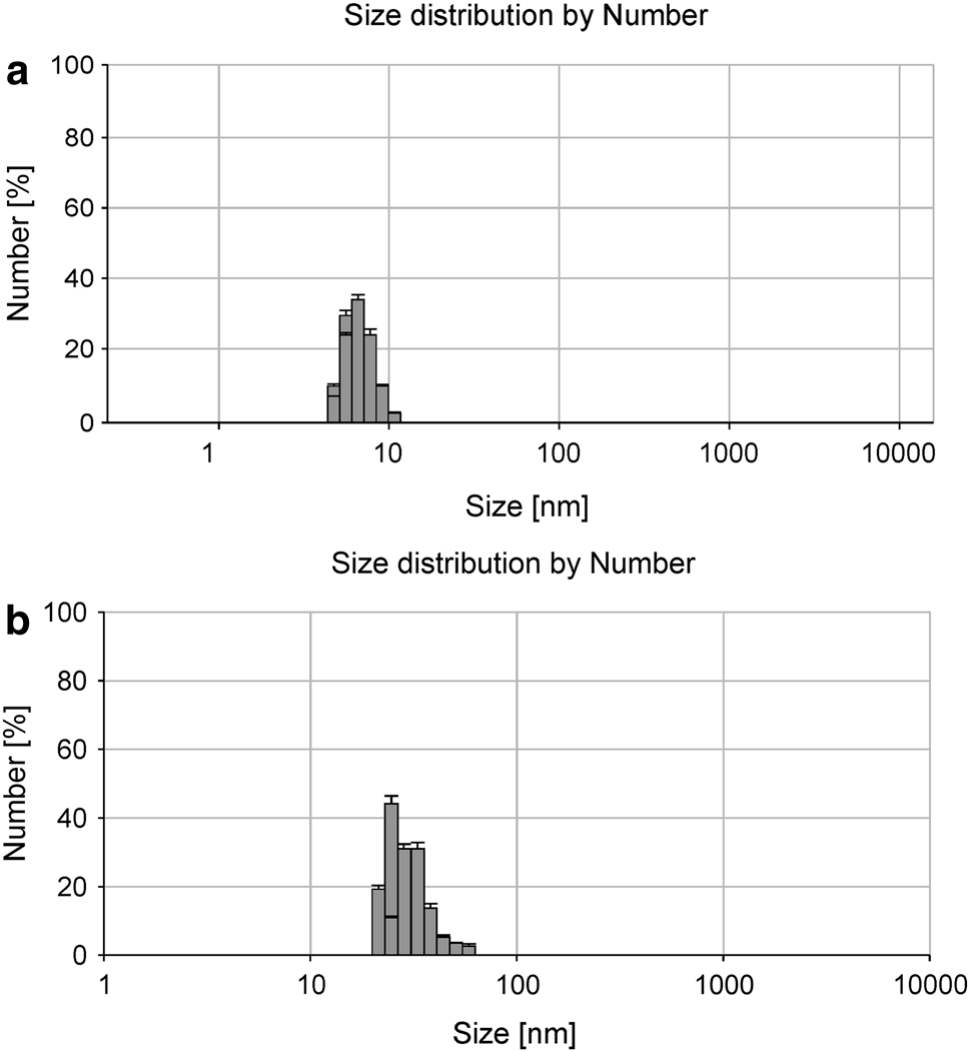
Typical size distribution of BDNF-PAMAM (**a**) and PEG-ylated BDNF-PAMAM (**b**) nanoparticles measured in the bulk by DLS (0.15 M PBS, pH 7.4) without an ultrafiltration process. All values are representative of 5 independent experiments and are expressed as mean ± SD

Dynamic light scattering analysis for BDNF-PAMAM nanoparticles revealed a mean diameter of 7.1 ± 1.1 nm, suggested by a relatively low polydispersity index (PDI) of less than 0.3. As shown in Fig. 2a, the mean hydrodynamic diameter value obtained for BDNF-PAMAM nanoparticles were significantly lower from the PEG-ylated BDNF-PAMAM ones (10.5 ± 1.3 nm), showing that PEG-functionalization influences the average size and polydispersity of the nanoparticles. The length of PEG in fully stretched conformation with molecular weight of 4 kDa gives 32 nm in length. Results obtained from DLS clearly indicate that PEG chains are coil, fold or twist on PAMAM-BDNF surface.

The electrophoretic mobility of BDNF-PAMAM nanoparticles (suspension of the bulk concentration 10 mgL^−1^ PAMAM and 0.02–2 mgL^−1^ BDNF) obtained a negative value of − 1.51 ± 0.35 μm cm/s V to − 1.72 ± 0.19 μm cm/s V, which corresponds to a zeta potential of − 19.2 ± 4.38 mV to − 22 ± 2.45 mV (calculated using the Henry relationship as described elsewhere) [48, 49] for pH 7.4; PBS. The zeta potential of BDNF-PAMAM-PEG nanoparticles (PEG bulk concentration is 25 mg L^−1^) increased with adsorption of polyelectrolyte layer and reached a value of -10.7 ± 2.2 mV, which indicates that the PEG molecule acquired a net of a negative charge. The negative zeta potential values obtained for above described nanoparticles were observed for unsaturated PEG layer. PEG modified the surface charge of BDNF-PAMAM nanoparticles; even though it is nominally uncharged, it reduces the number of charge groups on the PAMAM-based nanoparticles surfaces and thus affects colloidal stability.

The AFM measurements allowed us to determine the size range of nanoparticles adsorbed on mica surfaces under the diffusion-controlled transport condition at pH 7.4 and an ionic strength 0.15 M.

**Fig. 3:**
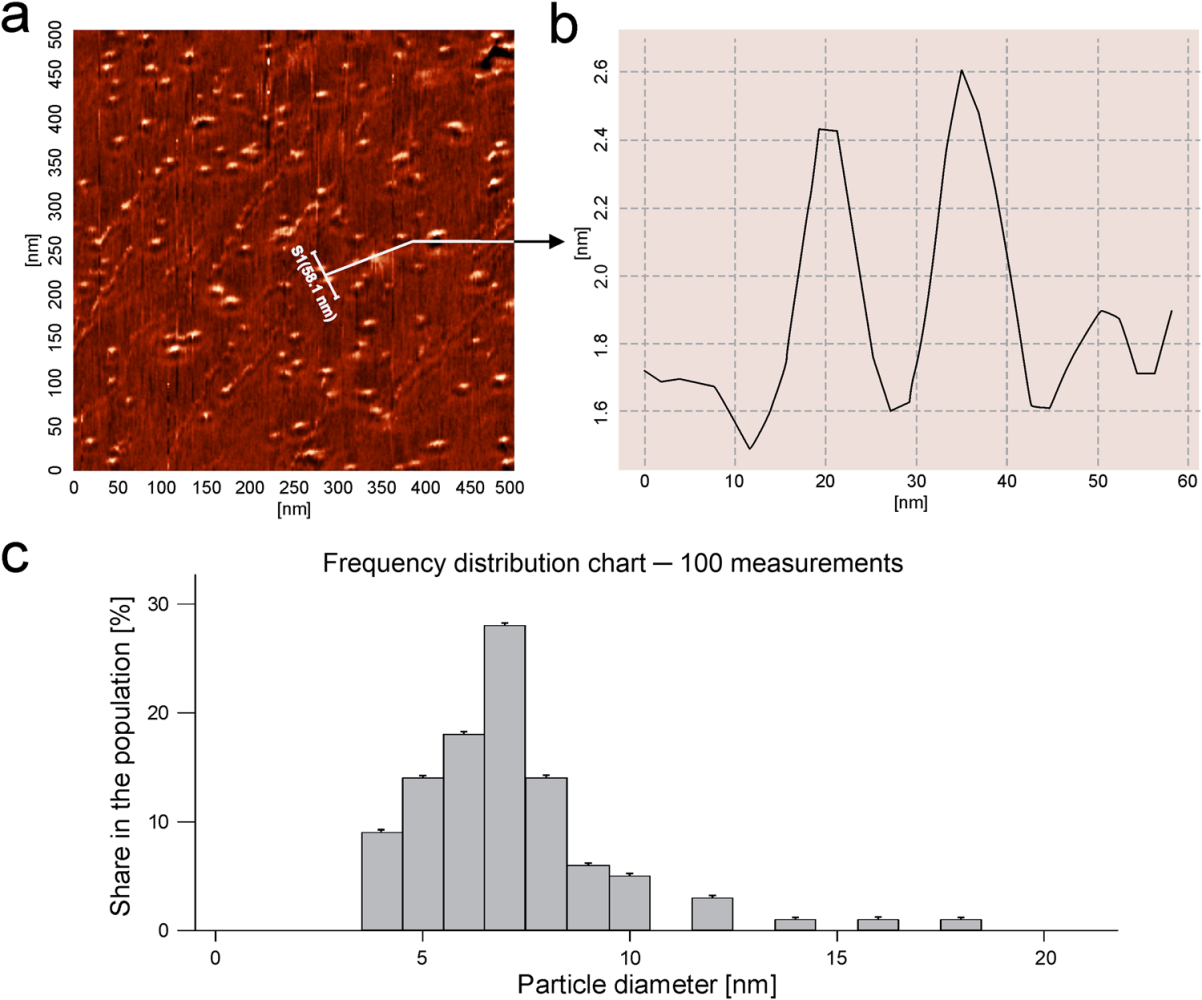

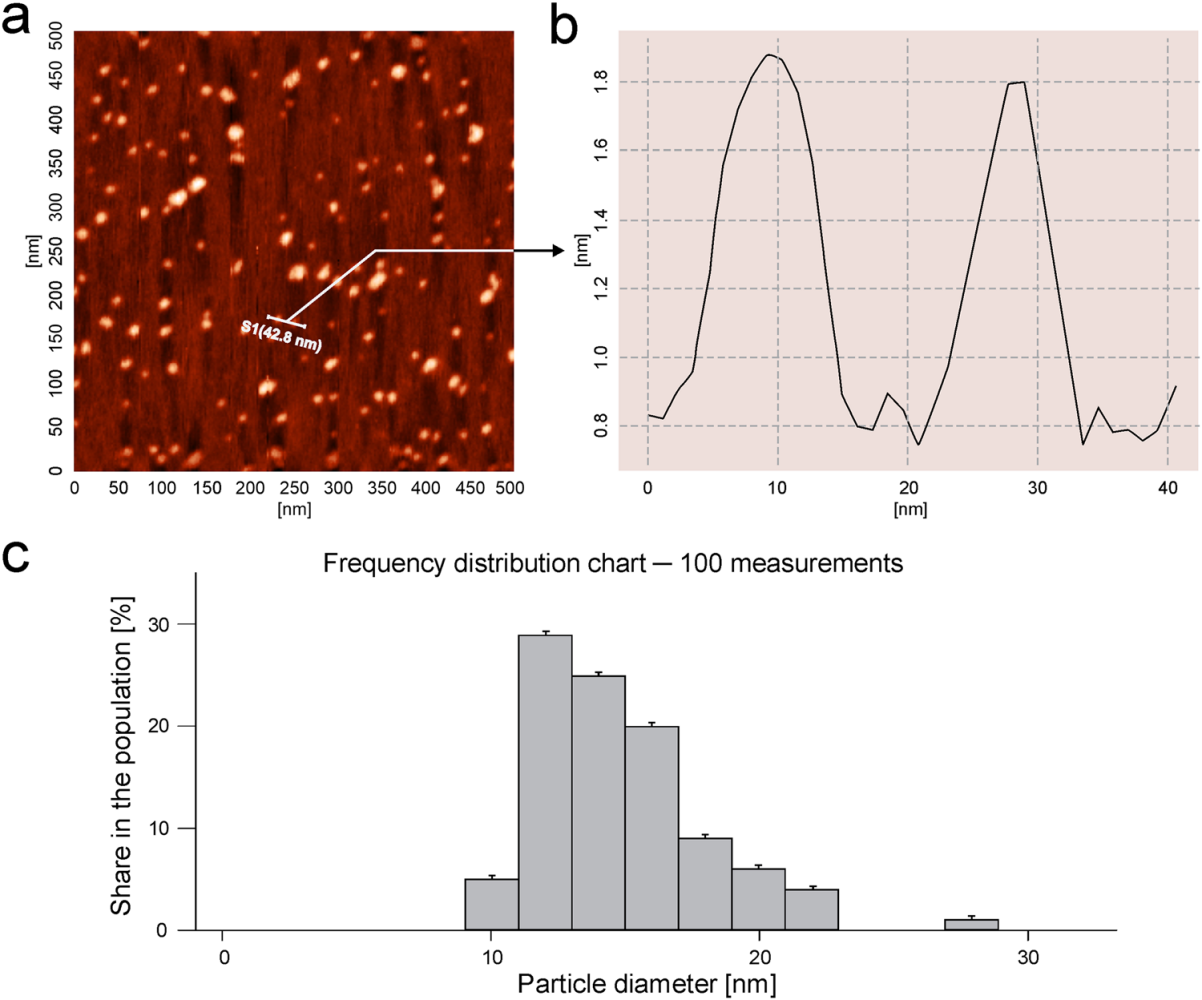
AFM analysis of PAMAM-BDNF (above part) and PEG-ylated PAMAM-BDNF (below part) nanoparticles adsorbed at mica surface at 0.15 M pH 7.4: PBS. **a** PAMAM-based nanoparticles at a scan area of 0.5 × 05 µm. **b** Structure of PAMAM-based nanoparticles after cross-section. **c** Histogram of adsorbed nanoparticles indicated by direct AFM enumeration, obtained for a low surface molecules concentration. The figure was created by taking into account 10 randomly chosen areas, where each micrograph of PAMAM-based monolayer at the mica surface has a size of 0.5 × 05 µm

As can be seen in Fig. 3, the average PAMAM-BDNF nanoparticle occupies the equivalent of spherical area with a diameter around 7 ± 2 nm. The distribution of PAMAM-BDNF nanoparticles diameters confirms that nanoparticles exist as isolated individuals. This enables us to exactly determine their sizes by cutting down the possibility of tip convolution artifacts. According to the results presented in Fig. 3, the size of the adsorbed single PAMAM-BDNF-PEG nanoparticle was around 11 ± 2 nm.

Moreover, colloidal stability of all PAMAM-based nanoparticles was probed by additional DLS studies. No significant size increase was found in PBS solutions of different protein concentrations, demonstrating the high colloidal stability of PEGylated BDNF-PAMAM nanoparticles. Contrary to BDNF-PAMAM nanoparticles, which show noticeable agglomeration tendency, PEGylation may improve colloidal nanoparticles stability via steric repulsion, even under high salt conditions.

### BDNF release from PAMAM-based nanoparticles in PBS buffer

In order to determine the possibility of desorption from dendrimers-based nanoparticles, once adsorption reached equilibrium, before the in vitro toxicity test was performed, we studied the BDNF release in PBS buffer.

Initially, adsorption of slightly positively charged BDNF molecules to negatively charged PAMAM dendrimers core surfaces was precisely determined to control the concentration of unbounded protein molecule and protein-laden nanoparticles structure. For every system the saturation concentration of protein has to be determined empirically. PAMAM nanoparticle is characterized with significant changes in its apparent zeta potential during adsorption, which can be efficiently monitored by LDV method. Importantly, at pH 7.4 BDNF molecules carry a net positive charge and adsorb onto negatively charged uniform surface. After loading of BDNF into PAMAM-based nanoparticles, we determined the dependence of the zeta potential of nanoparticles on the initial concentration of BDNF in the PAMAM suspension (after mixing). As is depicted in Additional file 1: Figure S1, zeta potential abruptly increases with increasing BDNF concentration and approaches the plateau values of -15 mV, which is considerably below the zeta potential of BDNF molecules determined in the bulk (5 mV at 0.15 M ionic strength). The electrophoretic mobility of BDNF-PAMAM complex is far from the value obtained for electrophoretic mobility of BDNF in the bulk (2 mgL^−1^ protein concentration), which corresponds to the formation of unsaturated neurotrophins structures on PAMAM cores.

**Fig. 4:**
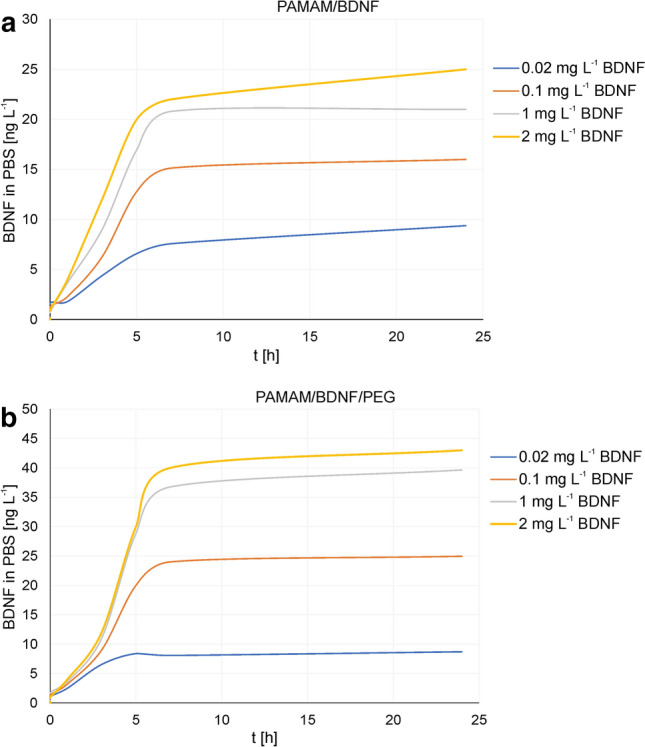
Desorption characteristics of BDNF from PAMAM G5.5 dendrimers-based nanoparticles in PBS electrolyte with increasing loading of protein concentrations from 0.02 to 1 mgL^−1^. BDNF detection by ELISA over 24 h incubation of (**a**) PAMAM-BDNF and (**b**) PEG-ylated PAMAM-BDNF nanoparticles

At this point, we applied ELISA method to evaluate more accurately concentration of desorbed BDNF molecules from PAMAM as well as PEG-PAMAM nanocarriers in electrolyte solution. The release profiles of BDNF molecules from nanoparticles at various initial concentration of protein from 0.02 to 2 mgL^−1^ is shown in Fig. 4 (pH 7.4, 0.15 M ionic strength, adsorption time 30 min).

For both type of nanoparticles, the first phase in releasing profile is characterized by a fast release of BDNF molecules from their surfaces, which probably results from the solubilization of protein adjoining dendrimer surface. In all cases, for BDNF loaded concentration of 0.02 mgL^−1^ the spontaneous electrostatic interaction led to the release of less than 10 ngL^−1^ of BDNF from 5 h up to 24 h, which gives less than 0.05% of loaded protein. One can see that for BDNF concentration lower than 0.02 mgL^−1^ at dendrimer surface, desorption of protein molecule was negligible, indicating that its adsorption onto 10 mgL^−1^ PAMAM nanoparticles was almost completed. This way we found that PAMAM molecules for laden BDNF concentration of less than 0.02 mgL^−1^ are likely to form irreversibly adsorbed BDNF layer, therefore the effect of substrate remains significant, making the desorption process less efficient than in case of densely packed layers.

For greater protein loadings, desorption of BDNF from PEG-PAMAM nanocarriers is characterized by a sustained release of protein molecules. For BDNF desorption kinetics from PEG-PAMAM nanoparticles, it can be noted that for the first 5 h we observed significant desorption of protein from nanoparticle surfaces in comparison to PAMAM-BDNF nanoparticles, indicating that additional polyelectrolyte layer improved the diffusion of the weakly entrapped protein from nanoparticles surfaces. For both types of nanocarriers, for the highest concentration of laden protein used, BDNF concentration reached maximum constant value after 10 h of desorption, for PAMAM-BDNF nanoparticles and PEG-ylated PAMAM-BDNF (Fig. 4) equal to 21 and 39 ngL^−1^, respectively. For higher protein concentration, BDNF molecules are forced to adsorb at a protein previously bound to the dendrimer surface, thus enhancing repulsive electrostatic interactions within the layers which cause the increase in binding energy of these molecules. These data strongly suggest that some fraction of less tightly bound BDNF molecule to PAMAM surface exists, which improves penetration across the PEG layer. Abruptly desorbed fraction of reversibly bound BDNF molecules from dendrimer-based nanocarrier during the course of desorption, may indicate the existence of certain population of neurotrophin aggregates in the adsorbed state or conformational changing of a single BDNF molecule during adsorption. This suggests that BDNF molecules reside within the PEG coating of the PAMAM rather than on the PAMAM surface.

Moreover, considering data obtained from ELISA and LDV measurements we noticed that irreversibility of BDNF adsorption process at dendrimers surfaces should be expected at pH significantly lower than isoelectric point of protein, where BDNF molecules and dendrimers particles have opposite electrokinetic charge. The BDNF desorption from PAMAM surfaces was consistent with previous data obtained for another neurotrophin (NT-4) desorbed from solid surface; results were interpreted in terms of the electrokinetic model for the concentration range of 0.1–1 mgL^−1^, postulating an irreversible adsorption of the protein governed by the random sequential adsorption.

### Nanoparticle toxicity

In order to determine the possibile toxicity of PAMAM-based nanoparticles in biomedical applications under increased concentration of BDNF, we examined cell viability in a differentiated neuroblastoma SH-SY5Y cells exposed to 6-OHDA. First, we differentiated SH-SY5Y cells by combination of RA treatment and lowering FBS in cell culture according to previous reports [44, 50–52]. We observed that extension of neurites, a typical neuronal phenotype, was visible 48 h after application of retinoic acid and retained till 7 days according to Ref. [44].

**Fig. 5:**
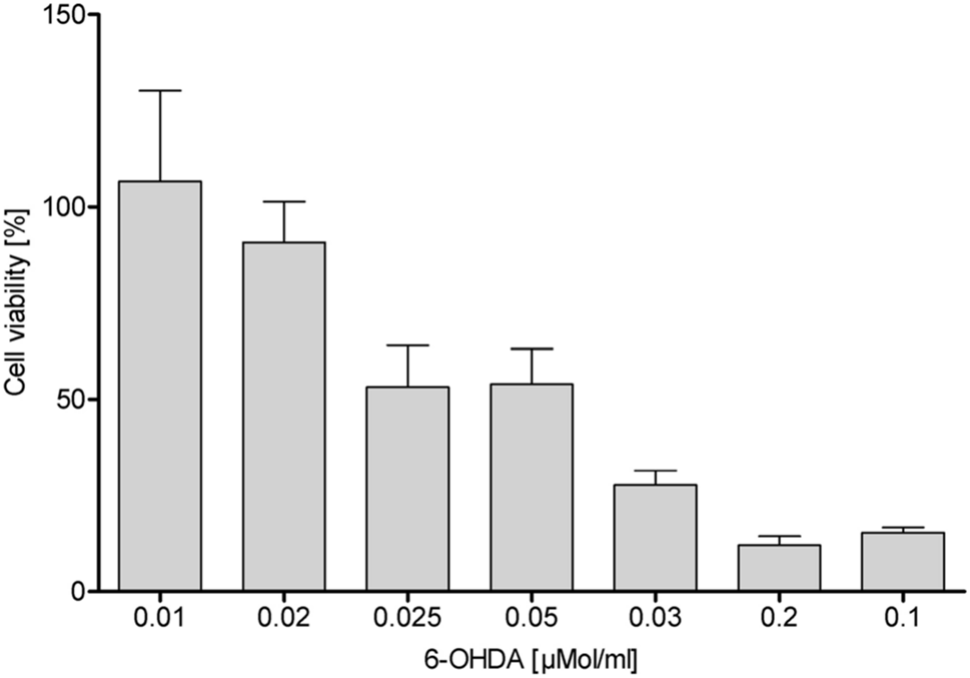
Differences in the sensitivity of differentiated SH-SY5Y cells to the 6-OHDA neurotoxin. The data represent means ± SD for 30 experiments

**Fig. 6:**
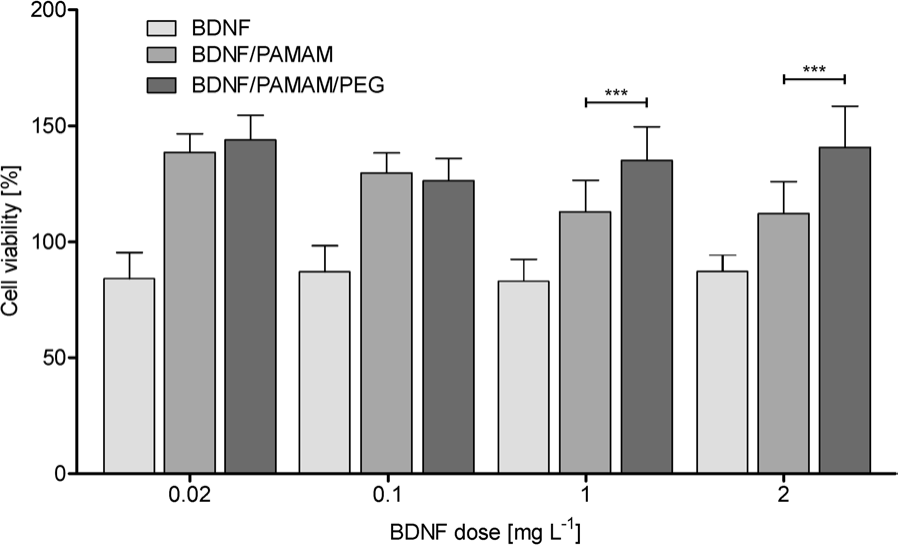
Cytotoxicity curves for BDNF, PAMAM-BDNF and PEG-ylated PAMAM-BDNF nanoparticles in differentiated human neuroblastoma cell line SH-SY5Y treated with the 100 µmol/L 6-OHDA neurotoxin. The control (100% viability) are cells treated with 100 µmol/L 6-OHDA only. The data represent means ± SD for 30 experiments

Systemic administration of 6-OHDA toxin is known to selectively impair the dopaminergic neurons, resulting in cell death and selective death of neurons in substantia nigra and striatum in animal models [53]. To establish experimental dosage for testing toxicity of PAMAM-based nanoparticles, we examined differentiated SH-SY5Y cells responsiveness to various concentrations of 6-OHDA for 24 h by MTT assay (Fig. 5). Reduction of cell viability was employed here as an indicator of cell proliferation and toxicity. We established that exposure to 100 µmol/L 6-OHDA resulted in a significant 70% decline in cell viability. After exploring the differences in toxicity upon exposure to varying concentrations of 6-OHDA, we chose 100 µmol/L 6-OHDA for further studies and used this concentration to determine the cytotoxicity of PAMAM-based nanoparticles with different loading BDNF concentration on differentiated SH-SY5Y cells after 24 h of incubation (Fig. 6).

For neuroblastoma cell line, the critical PAMAM dendrimer concentration, above which significant decrease in cell viability (cytotoxicity) occurs is ∼20 mg L^−1^ (Additional file 1). The MTT assay demonstrated that no observable toxicity was detected for nanoparticles with 10 mgL^−1^ concentration of PAMAM 5.5 dendrimers. The data presented in Fig. 6 further shows that after 24 h of nanoparticles incubation with SH-SY5Y, the cell viability increases from initial value obtained for BDNF without any carriers (90%) to ~ 150% in case of PAMAM-based nanoparticles. Moreover, with increasing concentration of protein loading, an increase in metabolic activity/cell number was observed only for PEG-PAMAM-BDNF nanoparticles, not for PAMAM-BDNF nanoparticles. We observed a marked increase in viability of SH-SY5Y cells treated with 100 µmol/L 6-OHDA in the presence of PAMAM-BDNF and PEG-PAMAM-BDNF nanoparticles for high protein concentration (41 and 52%, respectively) compared with cells treated with BDNF without carrier. This indicates that BDNF loading into PAMAM/PEG markedly increases in vitro cellular viability in the presence of neurotoxin.

### Cellular uptake and imaging of PAMAM-based nanoparticles in neuroblastoma cell line SH-SY5Y treated with 6-OHDA

To understand how PAMAM-BDNF and PEG-PAMAM-BDNF nanoparticles behave in neural-like cellular environment previously exposed to neurotoxing, we used confocal microscopy as well as spectrofluorimetry. Confocal analysis was conducted to qualitatively assess the localisation of BDNF-PAMAM-AF488 and BDNF-PAMAM-AF488-PEG nanoparticles and cellular uptake up to 24 h.

**Fig. 7:**
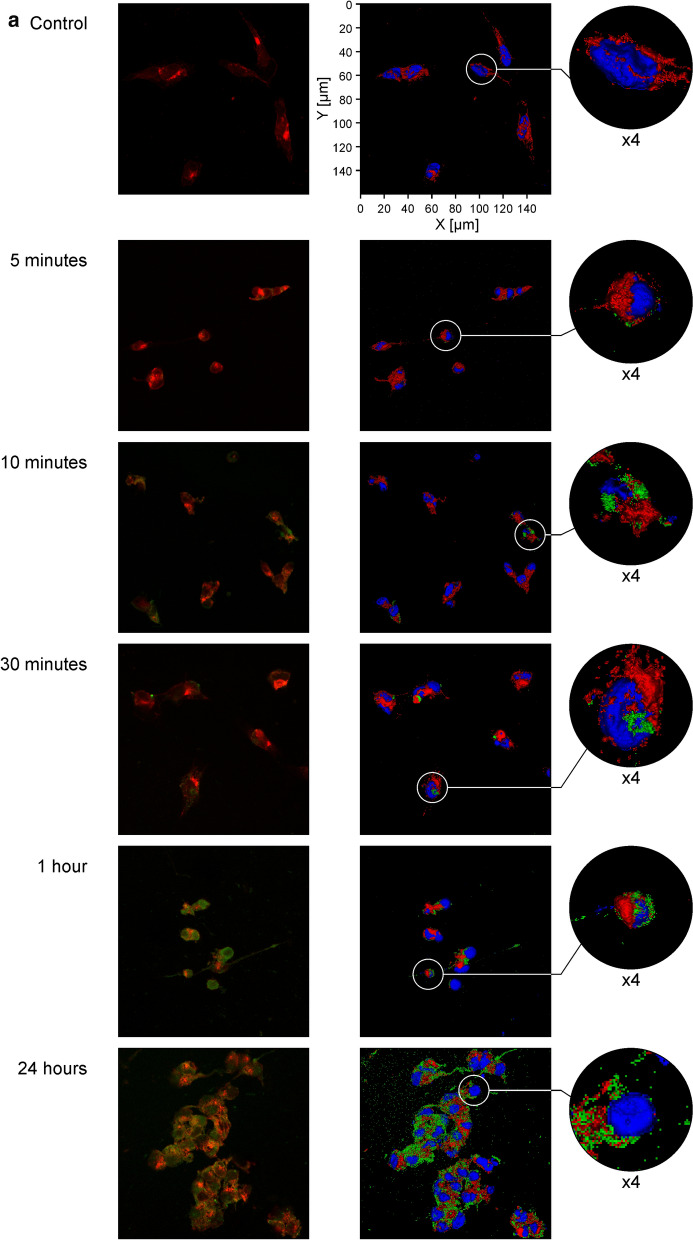

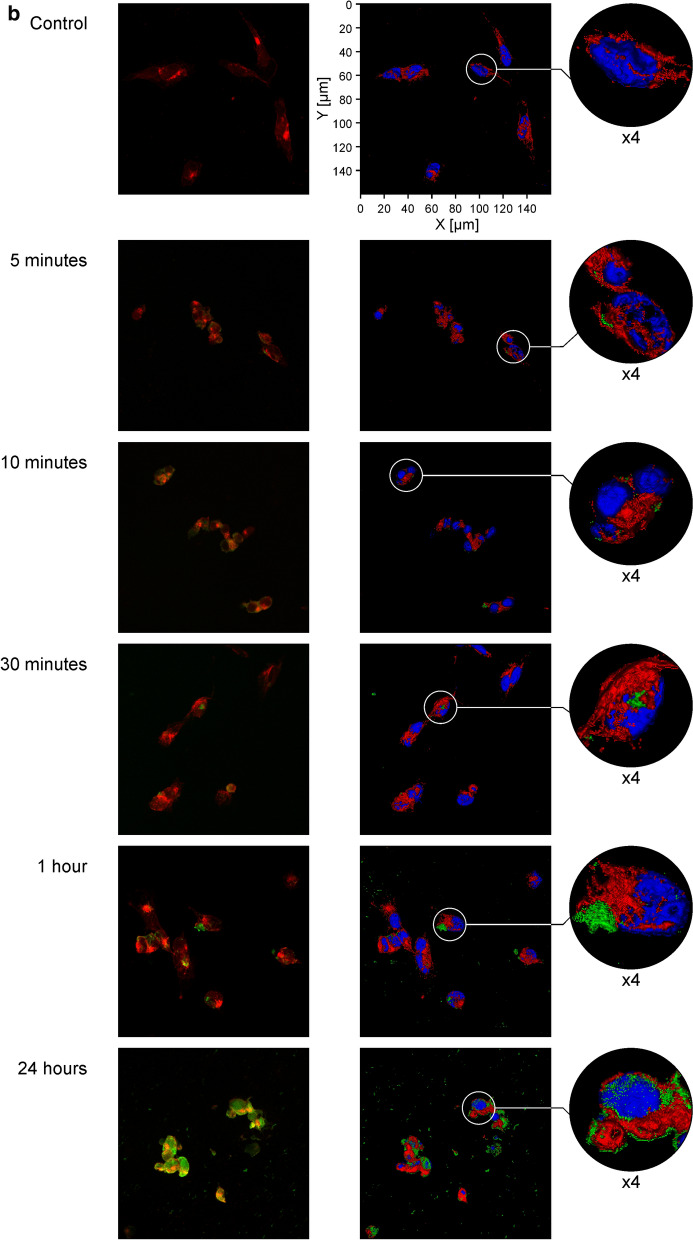
Cellular localisation of PAMAM-based nanoparticles in SH-SY5Y cells exposed to 6-OHDA. The cells were incubated either with BDNF-PAMAM-AF488 (**a**) or BDNF-PAMAM-AF488-PEG (**b**) nanoparticles (green color) for different time (5, 10, 30 min, 1 and 24 h) and then co-stained with WGA-Texas Red-X (red) and DAPI (blue). Confocal fluorescence images on the right panel are render series of z-stack with applied surface mode. Panel on the left presents single stack from the z-stack and only nanoparticles (green) and surface glycoproteins (red) are shown. The images were taken at 400 × magnification

As shown in Fig. 7, it seems that both types of nanoparticles are rather coated on SH-SY5Y cell surface than internalized. We found that from 5 min after the addition of PAMAM based nanoparticles with 0.1 mgL^−1^ BDNF concentration to up to 24 h, there was no observable cellular internalization, likely because of inefficient endocytosis. These results were more apparent for BDNF-PAMAM-AF488-PEG nanoparticles, which strongly suggests that protein molecules encapsulated in polyelectrolyte with their appropriate sizes are well protected and effectively adsorbed on cells membrane. PEG’s floppy chains and their charge neutrality can prevent non-specific adsorption and prevent or enhance linking chemistry via electrostatic repulsion or attraction, this involves nanoparticles interactions with an appropriate number of cell surface sites, which are necessary to produce an adequate binding energy.

In addition, nanoparticles with BDNF were incubated with SH-5YSY neuroblastoma cells for a different time (2, 5, 10, 30 min, 1, 4 and 24 h) and subjected to examinations by spectrofluorimetry evaluation. The cellular localisation of BDNF-PAMAM-AF488 and BDNF-PAMAM-AF488-PEG in SH-5YSY neuroblastoma cells was determined by green fluorescence intensity.

In line with the confocal microscopy results, significantly more pronounced green fluorescence was observed within the cells treated with BDNF-PAMAM-AF488 compared to BDNF-PAMAM-AF488-PEG. This confirms significantly hampered cellular internalization, likely because of inefficient endocytosis of nanoparticles by cells. It seems that the nanoparticles are bound to the cellular membrane. Due to the limitations of spectrofluorimetry method, we are unable to unequivocally assess whether nanoparticles enter the cells or not.

**Fig. 8:**
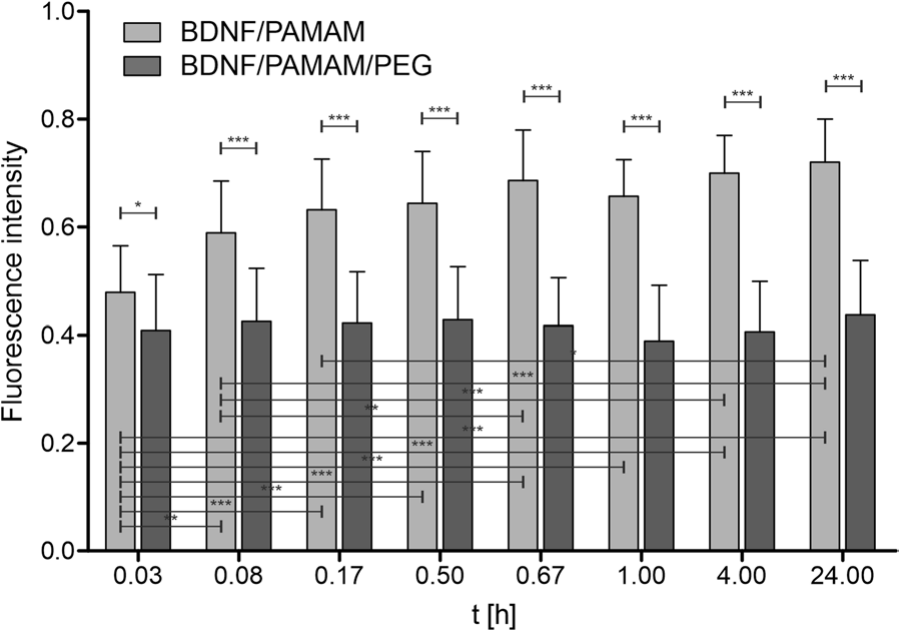
Immunofluorescence assessment of dendrimers-based nanoparticles localisation in neuron-like differentiated human neuroblastoma cell line SH-SY5Y upon treatment for 0.2–60 min, 4 and 24 h. Data is presented as the mean ± SD (n = 12). Data distribution was tested using Shapiro–Wilk test. To compare two analyzed datasets the unpaired t-test was used. The analysis of variances between different time-points was performed using one-way ANOVA. *p < 0.05, **p < 0.01, ***p < 0.001

As shown in Fig. 8, upon incubation of SH-5YSY neuroblastoma cells with BDNF-PAMAM-AF488, an increase of green fluorescence was evident after 24 h, in a dramatic contrast to that of 2 min-treated cells where little green fluorescence was observed, suggesting an efficient interaction with cell membrane for both types of nanoparticles. In fact, given enough time, more BDNF molecules could be released from PAMAM-based nanoparticles in immediate vicinity and subsequently enter the cell. Cellular uptake in serum-free media seems to be greatly reduced for PEG-ylated PAMAM-BDNF nanoparticles compared to the non-PEGy-lated ones.

### BDNF release from PAMAM-based nanoparticles in vitro

**Fig. 9:**
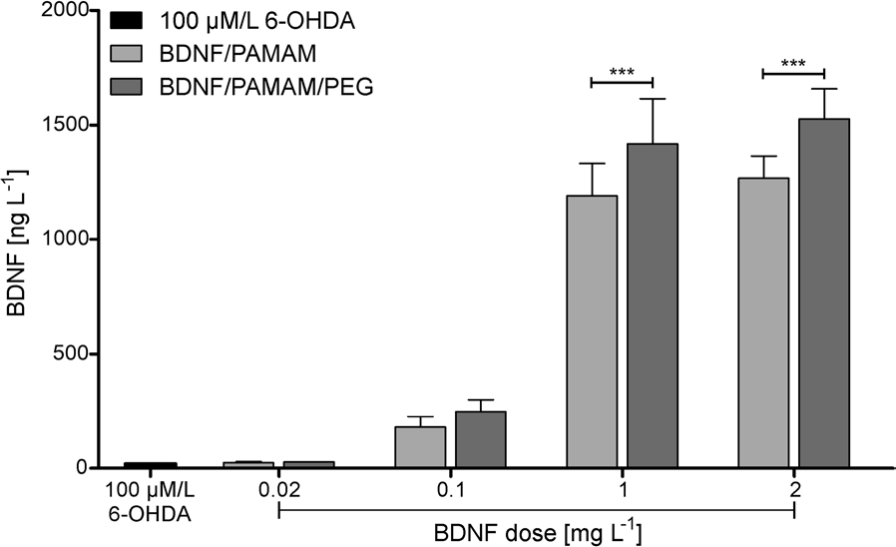
Desorption characteristic of BDNF from PAMAM G5.5 dendrimers-based nanoparticles for cell culture supernatant under increasing nanocarriers loading of protein concentrations from 0.02 to 2 mgL^−1^. BDNF detection by ELISA over 24 h in cells incubated with BDNF, PAMAM-BDNF nanoparticles and PEG-ylated PAMAM-BDNF nanoparticles. The data represent means ± SD for five experiments

Next, we determined whether BDNF from dendrimer-based nanoparticles is sustainably delivered. We investigated the suitability of PAMAM nanoparticles to effectively transport BDNF to differentiated neuroblastoma cells exposed to 6-OHDA. To assess the distribution of applied dendrimer-neurotrophin conjugates, we analyzed BDNF concentrations in cell culture supernatant after administration of BDNF and nanoparticles, using ELISA after 24 h post-treating. The cells exposed to RA and 25 µmol/L 6-OHDA served as controls. The results are summarized in Fig. 9.

We observed that BDNF concentration in the PAMAM-based nanoparticles-treated groups significantly increased compared to the control at 24 h. Released BDNF from PAMAM-based nanoparticles to extracellular region led to a significant increase in its concentration level compared to the control groups (100 µM 6-OHDA) for both type of PAMAM-based nanoparticles. We found that PEG-ylated PAMAM-BDNF nanoparticles-treated cells expressed significantly higher level of BDNF when compared with the PAMAM-BDNF nanoparticles-treated group. These difference in the BDNF levels in extracellular region in the PEG-ylated PAMAM-BDNF nanoparticles-treated group compared to the PAMAM-BDNF nanoparticles-treated group are in line with releasing protein profile presented in PBS. For BDNF release kinetics from PEG-BDNF-PAMAM nanoparticles in PBS after 24 h of treating, we observed significant desorption of BDNF from nanoparticle surfaces compared to PAMAM-BDNF nanoparticles of laden protein of 1 and 2 mgL^−1^ equal to 39 and 42 ngL^−1^, respectively. Treating differentiated SH-SY5Y cells with PAMAM-based nanocarriers intensifies the secretion of BDNF. These data strongly suggest that there is some interaction between surface TrkB cell receptors and BDNF nanoparticles in that system. Thus, the local secretion of BDNF may exert its action locally, through its stimulatory effect on nerve regeneration acting as a retrograde signal for survival neurons via the TrkB receptor. The increase in size, colloidal stability and reduction of surface charge density for PEG-ylated nanoparticles could lead to less efficient cellular uptake by differentiated SH-SY5Y cells treated with 6-OHDA through hampering interactions with their TrkB receptors.

## Conclusion

In the design of nanocarriers for neurodegenerative diseases treatment the sustained delivery of neuroprotective protein is desirable. Therefore, in this study we introduced a versatile nanoparticles based on the use of dendrimers for BDNF delivery.

Rapid cellular interaction of PAMAM-BDNF nanoparticles with cell membranes was detected as early as 2 min post-treatment, which could positively affect BDNF release for neurotoxin-injured cells at later time points. Encapsulation of PAMAM-BDNF nanoparticles into PEG layer further improves the design of the nanoparticle, through increasing colloidal stability and BDNF release. Increase in concentration of released BDNF in extracellular region as well as evidently enhanced cellular viability for PEG-ylated PAMAM-BDNF nanoparticles-treated group compared to the PAMAM-BDNF nanoparticles-treated group in differentiated SH-SY5Y cells treated with 6-OHDA strongly suggest a lower insertion of PEGylated nanoparticle into intracellular environment. PEGylated PAMAM-based nanoparticles could possibly allow us to enhance therapeutic efficacy of delivered proteins in neuron-like cells and devise a robust procedure for preparing stable and well-controlled dendrimer-based nanoparticles.

## Supporting information

The dependence of the zeta potential of PAMAM 5.5 nanoparticles on the initial BDNF concentration in the suspension at pH 7.4, 0.15 M ionic strength. Determination of the cytotoxicity of PAMAM 5.5 nanoparticles on differentiated human neuroblastoma SH-SY5Y cells exposed to 6-OHDA after 24 h of incubation.

## Supplementary information

**Additional file 1: Figure S1.** The dependence of the zeta potential of PAMAM 5.5 dendrimers on the initial BDNF concentration in the suspension. **Figure S2.** The dependence of the zeta potential of negatively charged latex particles on the initial BDNF concentration in the suspension *c*_*BDNF*_ in PBS, pH 7.4, 0.15 M ionic strength. **Figure S3.** Cytotoxicity curves for various concentration of PAMAM 5.5 in differentiated human neuroblastoma cell line SH-SY5Y treated with (**a**) 100 µmol/L 6-OHDA, (**b**) 15 µmol/L 6-OHDA.

## Data Availability

The data required to reproduce these findings are availability for any research.

## References

[1] Tanila H (2017). The role of BDNF in Alzheimer's disease. Neurobiol Dis.

[2] Tome D, Fonseca CP, Campos FL, Baltazar G (2017). Role of neurotrophic factors in parkinson's disease. Curr Pharm Des.

[3] Baumert B, Sobuś A, Gołąb-Janowska M, Ulańczyk Z, Paczkowska E, Łuczkowska K, Zawiślak A, Milczarek S, Osękowska B, Meller A, Machowska-Sempruch K (2020). Local and systemic humoral response to autologous lineage-negative cells intrathecal administration in ALS patients. Int J Mol Sci.

[4] Ibanez CF (1995). Neurotrophic factors: from structure-function studies to designing effective therapeutics. Trends Biotechnol.

[5] Robinson RC, Radziejewski C, Stuart DI, Jones EY (1995). Structure of the brain-derived neurotrophic factor/neurotrophin 3 heterodimer. Biochemistry.

[6] Scott-Solomon E, Kuruvilla R (2018). Mechanisms of neurotrophin trafficking via Trk receptors. Mol Cell Neurosci.

[7] Bailey JJ, Kaiser L, Lindner S, Wust M, Thiel A, Soucy JP, Rosa-Neto P, Scott PJH, Unterrainer M, Kaplan DR, Wangler C, Wangler B, Bartenstein P, Bernard-Gauthier V, Schirrmacher R (2019). First-in-Human Brain Imaging of [(18)F]TRACK, a PET tracer for Tropomyosin Receptor Kinases. ACS Chem Neurosci.

[8] Chao MV (2003). Neurotrophins and their receptors: a convergence point for many signalling pathways. Nat Rev Neurosci.

[9] Li J, Zhang X, Tang X, Xiao W, Ye F, Sha W, Jia Q (2020). Neurotrophic factor changes are essential for predict electroconvulsive therapy outcome in schizophrenia. Schizophr Res.

[10] Zhou C, Zhong J, Zou B, Fang L, Chen J, Deng X, Zhang L, Zhao X, Qu Z, Lei Y, Lei T (2017). Meta-analyses of comparative efficacy of antidepressant medications on peripheral BDNF concentration in patients with depression. PLoS ONE.

[11] Atasoy IL, Dursun E, Gezen-Ak D, Metin-Armagan D, Ozturk M, Yilmazer S (2017). Both secreted and the cellular levels of BDNF attenuated due to tau hyperphosphorylation in primary cultures of cortical neurons. J Chem Neuroanat.

[12] Dravid A, Parittotokkaporn S, Aqrawe Z, O'Carroll SJ, Svirskis D (2020). Determining Neurotrophin Gradients in Vitro To Direct Axonal Outgrowth Following Spinal Cord Injury. ACS Chem Neurosci.

[13] BDNF Study Group (1999). A controlled trial of recombinant methionyl human BDNF in ALS: The BDNF Study Group (Phase III). Neurology.

[14] Geral C, Angelova A, Lesieur S (2013). From molecular to nanotechnology strategies for delivery of neurotrophins: emphasis on brain-derived neurotrophic factor (BDNF). Pharmaceutics.

[15] Lopes CDF, Goncalves NP, Gomes CP, Saraiva MJ, Pego AP (2017). BDNF gene delivery mediated by neuron-targeted nanoparticles is neuroprotective in peripheral nerve injury. Biomaterials.

[16] Dabkowska M, Roginska D, Klos P, Sobus A, Adamczak M, Litwinska Z, Machalinska A, Machalinski B (2019). Electrostatic complex of neurotrophin 4 with dendrimer nanoparticles: controlled release of protein in vitro and in vivo. Int J Nanomed.

[17] Stasko NA, Johnson CB, Schoenfisch MH, Johnson TA, Holmuhamedov EL (2007). Cytotoxicity of polypropylenimine dendrimer conjugates on cultured endothelial cells. Biomacromol.

[18] Parimi S, Barnes TJ, Callen DF, Prestidge CA (2010). Mechanistic insight into cell growth, internalization, and cytotoxicity of PAMAM dendrimers. Biomacromol.

[19] Feliu N, Kohonen P, Ji J, Zhang Y, Karlsson HL, Palmberg L, Nystrom A, Fadeel B (2015). Next-generation sequencing reveals low-dose effects of cationic dendrimers in primary human bronchial epithelial cells. ACS Nano.

[20] McNerny DQ, Leroueil PR, Baker JR (2010). Understanding specific and nonspecific toxicities: a requirement for the development of dendrimer-based pharmaceuticals. Wiley Interdiscip Rev Nanomed Nanobiotechnol.

[21] Li N, Cai H, Jiang L, Hu J, Bains A, Gong Q, Luo K, Gu Z (2017). Enzyme-sensitive and amphiphilic PEGylated dendrimer-paclitaxel prodrug-based nanoparticles for enhanced stability and anticancer efficacy. ACS Appl Mater Interfaces.

[22] Iezzi R, Guru BR, Glybina IV, Mishra MK, Kennedy A, Kannan RM (2012). Dendrimer-based targeted intravitreal therapy for sustained attenuation of neuroinflammation in retinal degeneration. Biomaterials.

[23] Lamy CM, Sallin O, Loussert C, Chatton JY (2012). Sodium sensing in neurons with a dendrimer-based nanoprobe. ACS Nano.

[24] Choi SK, Myc A, Silpe JE, Sumit M, Wong PT, McCarthy K, Desai AM, Thomas TP, Kotlyar A, Holl MM, Orr BG, Baker JR (2013). Dendrimer-based multivalent vancomycin nanoplatform for targeting the drug-resistant bacterial surface. ACS Nano.

[25] Wang F, Zhang B, Zhou L, Shi Y, Li Z, Xia Y, Tian J (2016). Imaging Dendrimer-grafted graphene oxide mediated anti-miR-21 delivery with an activatable luciferase reporter. ACS Appl Mater Interfaces.

[26] Cao J, Ge R, Zhang M, Xia J, Han S, Lu W, Liang Y, Zhang T, Sun Y (2018). A triple modality BSA-coated dendritic nanoplatform for NIR imaging, enhanced tumor penetration and anticancer therapy. Nanoscale.

[27] Kim K, Lee J, Jo G, Shin S, Kim JB, Jang JH (2016). Dendrimer-capped gold nanoparticles for highly reliable and robust surface enhanced raman scattering. ACS Appl Mater Interfaces.

[28] Ma W, Fu F, Zhu J, Huang R, Zhu Y, Liu Z, Wang J, Conti PS, Shi X, Chen K (2018). (64)Cu-Labeled multifunctional dendrimers for targeted tumor PET imaging. Nanoscale.

[29] Giri J, Diallo MS, Simpson AJ, Liu Y, Goddard WA, Kumar R, Woods GC (2011). Interactions of poly(amidoamine) dendrimers with human serum albumin: binding constants and mechanisms. ACS Nano.

[30] Neira JL, Correa J, Rizzuti B, Santofimia-Castano P, Abian O, Velazquez-Campoy A, Fernandez-Megia E, Iovanna JL (2019). Dendrimers as competitors of protein-protein interactions of the intrinsically disordered nuclear chromatin protein NUPR1. Biomacromol.

[31] Curtis RA, Ulrich J, Montaser A, Prausnitz JM, Blanch HW (2002). Protein-protein interactions in concentrated electrolyte solutions. Biotechnol Bioeng.

[32] Lin YL, Khanafer K, El-Sayed ME (2010). Quantitative evaluation of the effect of poly(amidoamine) dendrimers on the porosity of epithelial monolayers. Nanoscale.

[33] Yang B, Xu H, Wang S, Cai M, Shi Y, Yang G, Wang H, Shan Y (2016). Studying the dynamic mechanism of transporting a single drug carrier-polyamidoamine dendrimer through cell membranes by force tracing. Nanoscale.

[34] Domenech R, Abian O, Bocanegra R, Correa J, Sousa-Herves A, Riguera R, Mateu MG, Fernandez-Megia E, Velazquez-Campoy A, Neira JL (2010). Dendrimers as potential inhibitors of the dimerization of the capsid protein of HIV-1. Biomacromol.

[35] Pelaz B, del Pino P, Maffre P, Hartmann R, Gallego M, Rivera-Fernandez S, de la Fuente JM, Nienhaus GU, Parak WJ (2015). Surface functionalization of nanoparticles with polyethylene glycol: effects on protein adsorption and cellular uptake. ACS Nano.

[36] Fahrlander E, Schelhaas S, Jacobs AH, Langer K (2015). PEGylated human serum albumin (HSA) nanoparticles: preparation, characterization and quantification of the PEGylation extent. Nanotechnology.

[37] Gon S, Santore MM (2011). Sensitivity of protein adsorption to architectural variations in a protein-resistant polymer brush containing engineered nanoscale adhesive sites. Langmuir.

[38] Alberio T, Colapinto M, Natale M, Ravizza R, Gariboldi MB, Bucci EM, Lopiano L, Fasano M (2010). Changes in the two-dimensional electrophoresis pattern of the Parkinson's disease related protein DJ-1 in human SH-SY5Y neuroblastoma cells after dopamine treatment. IUBMB Life.

[39] Alberio T, Lopiano L, Fasano M (2012). Cellular models to investigate biochemical pathways in Parkinson's disease. Febs j.

[40] Faridi A, Yang W, Kelly HG, Wang C, Faridi P, Purcell AW, Davis TP, Chen P, Kent SJ, Ke PC (2019). Differential roles of plasma protein corona on immune cell association and cytokine secretion of oligomeric and fibrillar beta-amyloid. Biomacromol.

[41] Ghadami SA, Chia S, Ruggeri FS, Meisl G, Bemporad F, Habchi J, Cascella R, Dobson CM, Vendruscolo M, Knowles TPJ, Chiti F (2020). Transthyretin inhibits primary and secondary nucleations of amyloid-beta peptide aggregation and reduces the toxicity of its oligomers. Biomacromol.

[42] Obeso JA, Rodriguez-Oroz MC, Goetz CG, Marin C, Kordower JH, Rodriguez M, Hirsch EC, Farrer M, Schapira AH, Halliday G (2010). Missing pieces in the Parkinson's disease puzzle. Nat Med.

[43] Dąbkowska M, Adamczyk Z, Cieśla M, Adamczak M, Bober J (2018). Lysozyme monolayers at polymer microparticles: electrokinetic characteristics and modeling. J Phys Chem C.

[44] Lopes FM, Schroder R, da Frota ML, Zanotto-Filho A, Muller CB, Pires AS, Meurer RT, Colpo GD, Gelain DP, Kapczinski F, Moreira JC, Fernandes Mda C, Klamt F (2010). Comparison between proliferative and neuron-like SH-SY5Y cells as an in vitro model for Parkinson disease studies. Brain Res.

[45] Lehmensiek V, Tan E-M, Liebau S, Lenk T, Zettlmeisl H, Schwarz J, Storch A (2006). Dopamine transporter-mediated cytotoxicity of 6-hydroxydopamine in vitro depends on expression of mutant alpha-synucleins related to Parkinson's disease. Neurochem Int.

[46] Xicoy H, Wieringa B, Martens GJM (2017). The SH-SY5Y cell line in Parkinson’s disease research: a systematic review. Mol Neurodegener.

[47] Xie HR, Hu LS, Li GY (2010). SH-SY5Y human neuroblastoma cell line: in vitro cell model of dopaminergic neurons in Parkinson's disease. Chin Med J (Engl).

[48] Roberts D, Keeling R, Tracka M, van der Walle CF, Uddin S, Warwicker J, Curtis R (2015). Specific ion and buffer effects on protein-protein interactions of a monoclonal antibody. Mol Pharm.

[49] Delgado AV, Gonzalez-Caballero F, Hunter RJ, Koopal LK, Lyklema J (2007). Measurement and interpretation of electrokinetic phenomena. J Colloid Interface Sci.

[50] Kovalevich J, Langford D (2013). Considerations for the use of SH-SY5Y neuroblastoma cells in neurobiology. Methods Mol Biol.

[51] Cheung YT, Lau WK, Yu MS, Lai CS, Yeung SC, So KF, Chang RC (2009). Effects of all-trans-retinoic acid on human SH-SY5Y neuroblastoma as in vitro model in neurotoxicity research. Neurotoxicology.

[52] Mohammadniaei M, Yoon J, Choi HK, Placide V, Bharate BG, Lee T, Choi JW (2019). Multifunctional nanobiohybrid material composed of Ag@Bi2Se3/RNA three-way junction/miRNA/retinoic acid for neuroblastoma differentiation. ACS Appl Mater Interfaces.

[53] Beal MF (2001). Experimental models of Parkinson's disease. Nat Rev Neurosci.

