# Supplementary material for: Novel design of (PEG-ylated)PAMAM-based nanoparticles for sustained delivery of BDNF to neurotoxin-injured differentiated neuroblastoma cells
Source: J Nanobiotechnology. 2020 Aug 31;18:120. doi: 10.1186/s12951-020-00673-8 (PMC7457365; doi:10.1186/s12951-020-00673-8)
Supplement: Supplementary file 1 — Additional file 1: Figure S1. The dependence of the zeta potential of PAMAM 5.5 dendrimers on the initial BDNF concentration in the suspension. Figure S2. The dependence of the zeta potential of negatively charged latex particles on the initial BDNF concentration in the suspension cBDNF in PBS, pH 7.4, 0.15 M ionic strength. Figure S3. Cytotoxicity curves for various concentration of PAMAM 5.5 in differentiated human neuroblastoma cell line SH-SY5Y treated with (a) 100 µmol/L 6-OHDA, (b) 15 µmol/L 6-OHDA. [file 12951_2020_673_MOESM1_ESM.docx]

**SUPPORTING INFORMATION**

# Novel design of (PEG-ylated) PAMAM-based nanoparticles for sustained delivery of BDNF to neurotoxin-injured differentiated neuroblastoma cells

# Maria Dąbkowska^1^*, Karolina Łuczkowska^2^, Dorota Rogińska^2^, Anna Sobuś^2^, Monika Wasilewska^3^, Zofia Ulańczyk^2^, Bogusław Machaliński^2^

*^1^Department of Medical Chemistry,* ***Pomeranian Medical University, Rybacka 1, 70-204*** *Szczecin, Poland*

*^2^ Department of General Pathology,* ***Pomeranian Medical University, Rybacka 1, 70-204*** *Szczecin, Poland*

*^2^Jerzy Haber Institute of Catalysis and Surface Chemistry Polish Academy of Sciences,*

*Niezapominajek 8, 30-239 Cracow, Poland*

*Corresponding author,

1. Determining the maximum coverage via the LDV method.

The adsorption of BDNF molecules at PAMAM 5.5 dendrimers was studied *in situ* using electrophoretic mobility/zeta potential (ζ) measurements supplemented by the LDV (Laser Doppler Velocimetry) depletion method. LDV method has been introduced by Adamczyk et al.^1-9^ and is based on the measurement of ζ-potential/microelectrophoretic mobility changes during adsorption of tested protein on model colloid particle.

Electrophoresis and LDV methods were the basis for performing a quantitative analysis of the adsorption of BDNF molecules at polymer nano- and micro-particles for the nanomolar concentration range. Adsorption of the BDNF molecule on PAMAM dendrimers was connected with significant changes in their apparent ζ-potential after adsorption (Fig. S1).


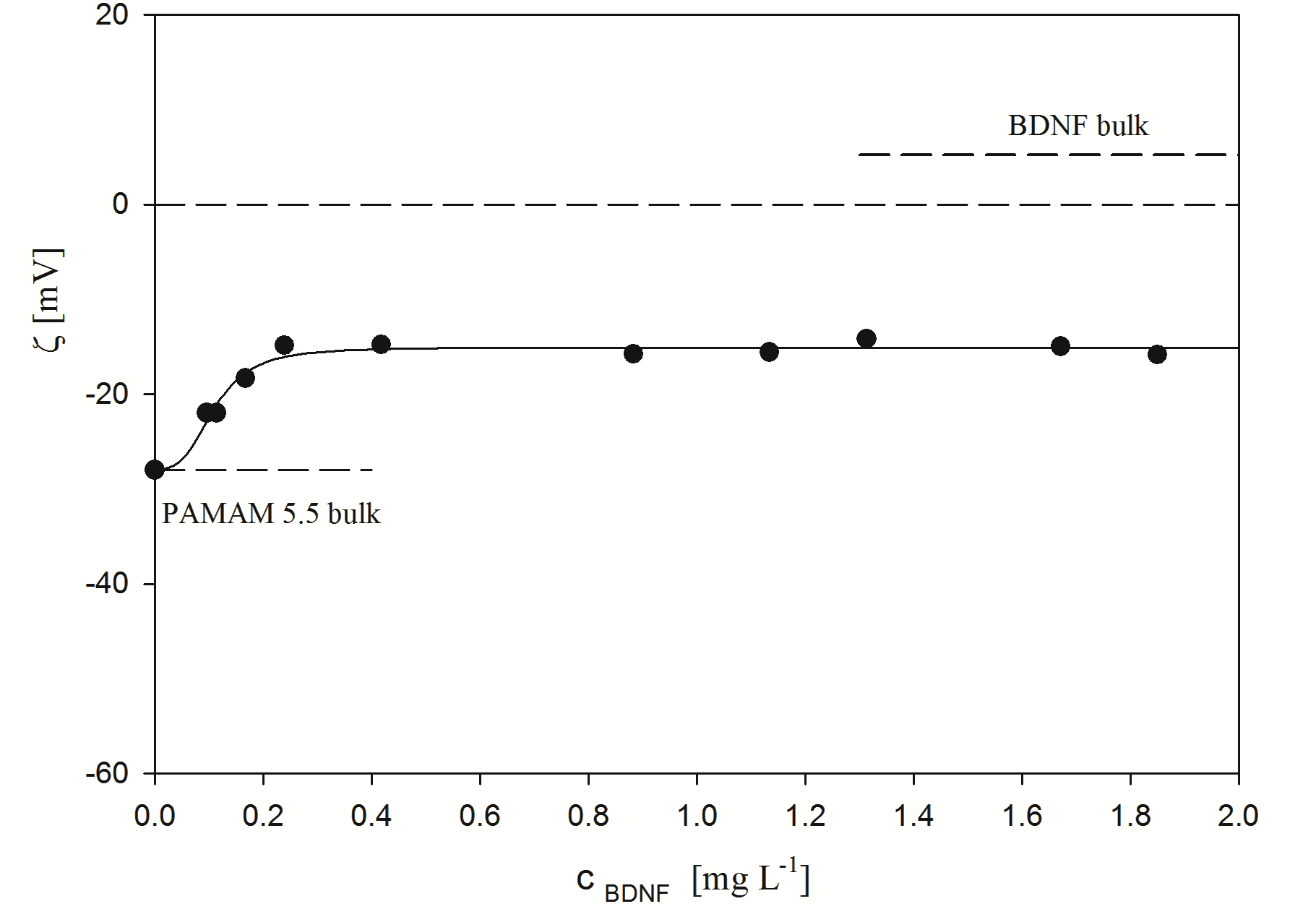


**Fig. S1.** The dependence of the zeta potential of PAMAM 5.5 dendrimers on the initial BDNF concentration in the suspension. *c_BDNF_*, PBS, pH 7.4, 0.15 M ionic strength.

As depicted in Figure S1, ζ-potential abruptly increased with increasing BDNF concentration and approached plateau values of −15 mV, which was far below the ζ-potential of BDNF in the bulk (5.2 mV at 0.15 M ionic strength). The electrophoretic mobility of the BDNF/PAMAM nanoparticles was much smaller than the electrophoretic mobility of bulk protein, which corresponded to the formation of an unsaturated BDNF layer on PAMAM for all protein concentrations used in the experiment.

To more accurately determine unbound BDNF after adsorption at PAMAM particles, two-step LDV method exploiting the calibrating measurements in order to determine protein maximum coverage at PAMAM nanoparticles was used. Laser Doppler Velocimetry method uses various colloid particles (in our study modelled latex microparticles) for efficient monitoring of desorbed protein molecule concentration. Initially, in these experiments, the dependence of the electrophoretic mobility of latex microparticles on the amount of BDNF added to the suspension was determined as a calibration curve (Figure S2 line 1). Afterwards, the PAMAM/protein mixture after the adsorption step is centrifuged in order to remove unbounded protein molecules and the supernatant is again contacted with bare latex particle suspension of known concentration (Figure S2 line 2). Using the LDV method, the electrophoretic mobility of the BDNF-latex particles complex is measured and converted to the bulk concentration of BDNF using calibration curves. The experimental results obtained by the LDV methods are presented in Fig. S2.

**
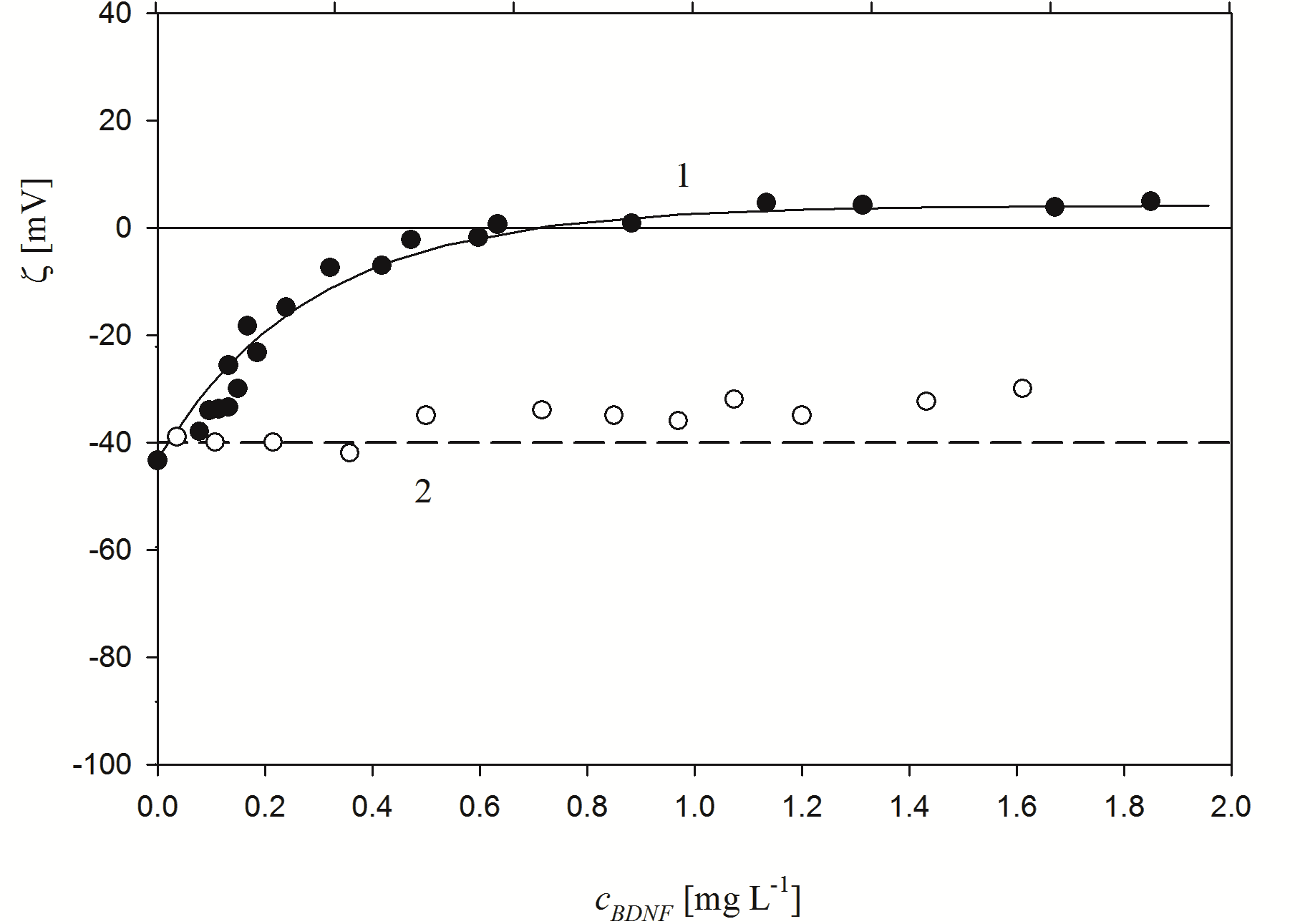
**

**Fig. S2.** The dependence of the zeta potential of negatively charged latex particles on the initial BDNF concentration in the suspension *c_BDNF_* in PBS, pH 7.4, 0.15 M ionic strength. The solid line 1 represents the theoretical results calculated from Eqs^7^. The points show the reference experimental data obtained for the initial adsorption step (full points); the dashed line 2 shows the fit of the experimental data obtained for the second adsorption step (empty triangles).

The results obtained by the two-step LDV method are shown in Figure S2 as the dependence of the zeta potential of latex microparticles on the initial BDNF concentration added in the first step for PBS electrolyte at a 0.15 M ionic strength (line 1). It can be noted that for low BDNF concentration up to 2 mgL^−1^, the change in zeta potential of latex microparticle determined in the second step is negligible, which indicates that the residual protein concentration after adsorption is smaller than 0.01 mg L^−1^. This method is more robust than the time-consuming ELISA test and becomes more accurate for smaller protein surface concentration than the maximum coverage of protein molecules on colloid particles.

1. Determining the cytotoxicity of PAMAM 5.5 with the MTT assay.

We chose 100 µmol/L dose of 6-OHDA, which resulted in a significant 70% decline in cell viability as well as 15 µmol/L dose of 6-OHDA, which resulted in damaging 20% of differentiated human neuroblastoma SH-SY5Y cells, and various concentrations of PAMAM 5.5 on differentiated cells after 24h of incubation for further cytotoxicity studies. The experimental results obtained from the MTT assay are presented in Fig. S3.

a)

b)

**Fig. S3.** Cytotoxicity curves for various concentration of PAMAM 5.5 in differentiated human neuroblastoma cell line SH-SY5Y treated with a) 100 µmol/L 6-OHDA, b) 15 µmol/L 6-OHDA. The data represent means +/- SD for 20 experiments.

References:

1. Nattich-Rak, M. ; Sadowska, M. ; Adamczyk, Z. ; Cieśla, M. ; Kąkol, M. Formation mechanism of human serum albumin monolayers on positively charged polymer microparticles. Colloids Surf. B: Biointerfaces 2017, 159, 929-936.
2. Kujda, M.; Adamczyk, Z.; Cieśla, M. Monolayers of the HSA dimer on polymeric microparticles-electrokinetic characteristics. Colloids and Surfaces B: Biointerfaces 2016, 148, 229–237.
3. Adamczyk, Z.; Nattich-Rak, M.; Dąbkowska, M.; Kujda-Kruk, M. Albumin adsorption at solid substrates: A quest for a unified approach. Journal of Colloid and Interface Science 2018, 514, 769–790.
4. Cieśla, M.; Nowak, A. Managing numerical errors in random sequential adsorption. *Surf. Sci.*  2016, 651, 182-186.
5. Adamczyk, Z.; Sadlej, K.; Wajnryb, E.; Nattich, M.; Ekiel-Jeżewska, M. L.; Bławzdziewicz, J. Streaming potential studies of colloid, polyelectrolyte and protein deposition. Adv. Colloid Interface Sci*.* 2010, *153*, 1–29.
6. Sadlej, K.; Wajnryb, E.; Bławzdziewicz, J.; Ekiel-Jeżewska, M. L.; Adamczyk, Z. Streaming current and streaming potential for particle covered surfaces: Virial expansion and simulations. J. Chem. Phys. 2009, *130*, 144706.
7. Dąbkowska, M.; Adamczyk, Z.; Cieśla, M.; Adamczak, M.; Bober, J. Lysozyme monolayers at polymer microparticles:elektrokinetic characteristic and modeling. J.Phys. Chem. C 2018, 122, 17846-17855.
8. Kujda, M.; Adamczyk, Z.; Cieśla, M. Monolayers of the HSA dimer on polymeric microparticles-electrokinetic characteristics. Colloids and Surfaces B: Biointerfaces 148 (2016) 229–237.
9. Bratek-Skicki, A.; Żeliszewska, P.; Adamczyk, Z.; Cieśla, M. Human Fibrinogen Monolayers on Latex Particles: Role of Ionic Strength. Langmuir 2013, 29, 3700–3710.
